# A Social-Ecological Approach to Understanding the Relationship between Cyberbullying Victimization and Suicidal Ideation in South Korean Adolescents: The Moderating Effect of School Connectedness

**DOI:** 10.3390/ijerph182010623

**Published:** 2021-10-11

**Authors:** Jungup Lee, JongSerl Chun, Jinyung Kim, Jieun Lee, Serim Lee

**Affiliations:** 1 Department of Social Work, National University of Singapore, Singapore 117570, Singapore; 2 Department of Social Welfare, Ewha Womans University, Seoul 03760, Korea; jschun@ewha.ac.kr (J.C.); kjj7260@hotmail.com (J.K.); jieun9433@gmail.com (J.L.); serim0507@naver.com (S.L.)

**Keywords:** cyberbullying victimization, suicidal ideation, South Korean adolescents, school connectedness, social-ecological approach

## Abstract

Background: Cyberbullying victimization and suicidal ideation are both ongoing deleterious social problems in South Korea. Using the social-ecological approach, this study examined the association between cyberbullying victimization and suicidal ideation as well as the buffering role of school connectedness in this relationship. Methods: A nationally representative sample of 7333 adolescents from the 2016 Korean Children and Youth Right Study participated in the study. Data were analyzed using descriptive statistics, Wald chi-square test, bivariate correlations, and multivariate logistic regression analyses. Results: Nearly 17.7% of adolescents were cyberbullied, and 28.4% had suicidal ideation in the past 12 months. Cyberbullying victims were at an increased risk of suicidal ideation. The results also found that parental abuse, family dysfunction, and perceived peer relationship stress were positively associated with suicidal ideation, while parental support for autonomy was negatively associated with suicidal ideation. Further, school connectedness moderated on the relationship between cyberbullying victimization and adolescent suicidal ideation. Conclusions: These findings suggest that various stakeholders should consider interventions and preventive programs that address school connectedness when working with adolescents who are victims of cyberbullying and exhibit suicidal behavior.

## 1. Introduction

In response to the rapid growth of technologies and increasing usage of smartphone and social media, cyberbullying is now a global concern. Cyberbullying victimization (CBV) is defined as being the target of aggressive or harmful behavior through any form of electronic communication device [1]. Cyberbullying takes place on social media, messaging and gaming platforms, and mobile phones, and it is generally committed repeatedly to those who are targeted [2]. UNICEF (2020) has presented examples of cyberbullying: “spreading lies about or posting embarrassing photos of someone on social media; sending hurtful messages or threats via messaging platforms; impersonating someone and sending mean messages to others on their behalf.” Cyberbullying and offline bullying often occur alongside; however, cyberbullying leaves some records [2]. The effects of cyberbullying victimization vary. UNICEF suggested that cyberbullying might affect victims mentally, emotionally, and also physically [2]. Moreover, a previous study in South Korea revealed six major impacts of cyberbullying victimization: internalized and externalized problems, school and peer problems, online problems, seeking social support, and avoidance [3].

In South Korea, 19% of school-aged children experienced CBV only, and 26.9% experienced CBV/cyberbullying perpetration (CBP) [4]. Being a cyberbullying victim during adolescence has long-lasting negative impacts on psychological, physical, and behavioral adjustment and results in social problems [3,5,6]. Studies indicate that Korean adolescent cyber victims report internalized problems as major impacts of their cyberbullying experience, and suicidal ideation and behaviors are the most deleterious effects of CBV [3,7]. Given existing evidence, more attention should be given to the relationship between CBV and suicidal behavior. The suicide rate per 100,000 adolescents aged 10–19 stood at 5.9 in 2019, up from 4.9 in 2016, and is still rising despite the efforts to reduce suicidal behaviors in society [8]. A recent study of 59,984 middle and high school students from the Korea Youth Risk Behavior Web-Based Survey (KYRBWS) reported that about 12.4% of adolescents had an episode of suicidal ideation/attempt [9].

Victims of cyberbullying are more likely to exhibit suicidal behavior. Indeed, an increasing number of studies have confirmed the association between CBV and suicidal behavior among the adolescent population. Research has shown that the risk of suicidal ideation among middle school students is heightened with more experiences in CBV [10]. Although suicidal behavior generally includes suicidal ideation, suicide attempts, and completed suicide, this study focuses primarily on suicidal ideation since it progressively connects to suicidal planning and suicide attempt [11], and it extends existing research into cyberbullying and suicidal ideation with Korean adolescents as its target group.

### 1.1. Suicidal Ideation from a Socio-Ecological Approach 

Many studies have revealed that female adolescents were more likely to report suicidal ideation than male adolescents [12,13]. In the association between CBV and suicidal ideation, female victims reported significantly higher levels of suicidal ideation than male victims. Regarding age, younger adolescents were more likely to attempt suicide than older adolescents [14], and middle school students showed higher suicidal ideation than high school students [15]. A low grade point average (GPA) score and low social-economic status (SES) also predicted higher levels of suicidal ideation among Korean adolescents [16]. Depression is one of the primary risk factors predicting suicidal ideation, while self-esteem is a vital protective factor for suicidal ideation among adolescents [17,18]. As for physical health, there was a negative relationship between subjective well-being and suicidal ideation among adolescents [13,19]

Within the family context, it had been consistently reported that family dysfunction and parental discord were risk factors for suicidality among adolescents [20,21]. Specifically, a broken family structure is not only associated with the initial level of suicidal ideation but also has a persistent impact over time [22]. Moreover, childhood abuse and neglect were associated with adolescent suicidal ideation and attempts [23,24]. Each type of child abuse (i.e., sexual, physical, and emotional abuse) showed an independent relationship with adolescent suicidal ideation and attempts; however, sexual and emotional abuse might be relatively more related to suicidality than physical abuse or neglect [24,25]. It is reported that sexual abuse has a stronger association with suicide ideation than physical abuse or witnessing violence [26]. In addition, it is suggested that although all types of child abuse affect adversely on child development, emotional abuse has especially detrimental and long-lasting effects on mental health and suicidality [27]. Autonomy can serve as a protective resource because it allows adolescents to develop their own effective coping strategies for handling life stressors. Indeed, adolescents’ psychological autonomy from parents served as a protective factor for suicidal ideation and psychological well-being [28,29].

Suicidal ideation among school-aged children is often associated with poor or negative peer relations [30]. Studies indicate peer relationship stress and peer conflict are significant factors that explain suicidal ideation and attempts among adolescents [31,32]. Additionally, several pieces of literature found that there was a strong association between peer victimization and suicidal ideation among adolescents and that the risk of suicidality varied according to the types of victimization as well as the levels of exposure to victimization [7,33,34].

Generally, the school plays a critical role in reducing and preventing adolescents’ suicidal ideation. A positive school climate and a sense of belonging to school buffered the relationship between CBV and suicidal ideation [35]. This implies that lower school support predicted a greater likelihood of suicidal ideation [36]. Besides the general role of the school itself, teachers also play a pivotal role in adolescents’ suicidal ideation: Engagement with teachers and teacher’s support were revealed to be inversely related to suicidal ideation [37]. 

### 1.2. Relationship between Cyberbullying Victimization and Suicidal Ideation

Cyberbullying victimization and suicidal ideation are both ongoing deleterious social problems in South Korea, and there is a growing body of literature that has examined the relationship between cyberbullying victimization and suicidal ideation in the last few years [38]. Prior studies have consistently demonstrated a relationship between CBV and suicidal ideation among adolescents [12,13]. Several meta-analyses found that cyberbullying was associated with suicidal ideation and even showed that this association was stronger than that between traditional bullying and suicidal ideation [7,39]. A study on youth violence and mental health for Asian and Pacific Islander students found that adolescent victims of cyberbullying were 3.2 times more likely to show suicidality compared with non-victimized counterparts [40]. Consistently, Korean studies also showed positive relationships between CBV and suicidal ideation [3,41]. The risk of suicidal ideation also increases with the severity level of CBV: While 35% of students suffering from a low level of CBV reported having suicidal ideation, this figure increased to 52.4% for those in the high-level group [42].

### 1.3. Moderating Effects of School Connectedness

School connectedness has been discussed as an important protective factor associated with suicidal ideation among adolescents and enhancing school connectedness could be a useful universal strategy for reducing suicidal ideation in adolescents [43,44]. A few studies have identified the interacting effect between school connectedness (i.e., ties, bond, and sense of belonging to schools) [45] and CBV on suicidal ideation, indicating that victims of cyberbullying who have more ties to school are less likely to think of or attempt suicide [46]. Similarly, feeling connected to an adult at school moderated the relationship between CBV and suicidal behaviors for sexual minority youth [47].

### 1.4. Current Study

We first sought to examine the relationship between CBV and suicidal ideation, considering the social-ecological covariates in South Korean adolescents. The social-ecological approach with individual-, family-, peer-, and school-level variables are imperative for the holistic understanding of the association between CBV and suicidal ideation. The second aim of the current study was to examine the effect of the interaction between social-ecological variables and CBV on suicidal ideation. According to the social-ecological model [48], adolescents are embedded in various interactive systems, including their family, peer, and school environment, and so, it is essential to understand youth problems such as suicidal ideation in the contexts of these social-ecological variables. The following hypotheses are formulated:

**Hypothesis** **1** **(H1).** *Being victims of cyberbullying would be associated with a higher risk of suicidal ideation.*

**Hypothesis** **2** **(H2).** *The individual, family, peer, and school variables of the social-ecological contexts would be associated with adolescent suicidal ideation.*

**H2-1.**  *In the individual context, female sex, younger, low GPA score, low SES, poor physical health, high depressive symptoms, and low self-esteem would be associated with a higher risk of suicidal ideation.*

**H2-2.**  *In the family context, parental abuse, parental neglect, and family dysfunction would be associated with a higher risk of suicidal ideation, whereas parental support would be associated with a lower risk of suicidal ideation.*

**H2-3.**  *In the peer context, peer victimization and perceived peer relationship stress would be associated with a higher risk of suicidal ideation.*

**H2-4.**  *In the school context, teacher abuse would be associated with a higher risk of suicidal ideation, while teacher support and school connectedness would be associated with a lower risk of suicidal ideation.*

**Hypothesis** **3** **(H3).** *School connectedness would moderate the relationship between cyberbullying victimization and suicidal ideation, with the association becoming weaker among adolescents with more school connectedness.*

## 2. Methods

### 2.1. Participants and Data Procedure

Data for the current study were retrieved from the Korean Children and Youth Right Study (KCYRS) in 2016. Conducted by the National Youth Policy Institute (NYPI), the KCYRS designed a cross-sectional, self-report, school-based public survey and included several components related to school violence and abuse, students’ perceptions of human rights and ethics, parent and peer relationship, health and disability, schoolwork, activity, and leisure among school-aged children and adolescents. The KCYRS used a multistage stratified cluster probability sampling to recruit a nationally representative sample of elementary, middle, and high school students in South Korea. A total of 393 schools across 16 provinces in South Korea were randomly selected by using the list of national schools collected by the Ministry of Education in 2015, and then student participants in the schools were randomly selected in proportion to sex and school types [49]. The final sample included 7333 Korean middle and high school students (equivalent to 7th to 12th graders in the United States). The average age of the study population was 15.39 (*SD* = 1.70, range 12–19). The participants consisted of 50.8% females, 48.5% in high school, 31.2% in low GPA, and 14.6% in low SES.

### 2.2. Instruments

#### 2.2.1. Cyberbullying Victimization

Cyberbullying victimization (CBV) was assessed with the Cyber Bullying Questionnaire (CBQ) developed by the Korean National Youth Policy Institute [50]. For this study, *CBV* included five items asking about the respondents’ incidents of CBV in the past 12 months: (a) being called mean names or being insulted, (b) being threatened, (c) being made the subject of sexual jokes, (d) being excluded from social community or chatroom online, and (e) personal information or private matters being widely spread. The response options were based on a 5-point scale ranging from “not at all” to “more than 3 times a week” (α = 0.65).

#### 2.2.2. Suicidal Ideation

Suicidal ideation was measured with a single item, “Have you ever thought about suicide in the past 12 months?”. The response options ranged from “never,” “sometimes,” and “often,” which were recoded dichotomously (no or yes).

#### 2.2.3. Social-Ecological Contexts

Social-ecological contexts included the individual-, family-, peer-, and school-level variables. The individual context consisted of age (in years), gender (male, female), school level (middle school, high school), school grade point average (GPA: low, middle, high), socio-economic status (SES: low, middle, high), and self-report physical health (very bad to very good). The adapted and shortened Korean version of the Child Behavior Checklist Youth Self-Report (K-CBCL) [51] was also used to assess both depressive symptoms (three items, including “lonely,” “nervous,” and “depressive”; not at all true to very true; α = 0.88) and self-esteem (four items; e.g., “I feel that I am a person of worth” and “I take a positive attitude toward myself”; not at all true to very true; α = 0.83).

For the family-level variables, parental abuse was measured with two items inquiring the extent of parents’ physical and verbal abuse in the past 12 months. Five response options ranged from “not at all” to “more than 3 times a week”. The Spearman–Brown coefficient for parental abuse was 0.66. Parental neglect was assessed with the three item, adapted Korean version of the Parenting Behavior Inventory (PBI) [52]. This scale inquired the extent of being neglected by parents in the past 12 months (e.g., “My parents leave me alone when I am sick”). Response options ranged from “not at all” to “more than 3 times a week” (α = 0.65). Parental support for autonomy was measured with four items asking about the level of parents’ respect and care for their child’s autonomy (e.g., “My parents respect my opinion when deciding on important family issues”). Response categories ranged from “not at all true” to “very true” (α = 0.84). Family dysfunction was indicated by a single item, “In the past 12 months, I have experienced high levels of stress as my family was dysfunctional” (not at all true to very true).

In the peer context, peer victimization was assessed with the Korean version of the Juvenile Peer Victimization Questionnaire (JPVQ) [50]. It included seven items asking the respondents if they had been bullied by peers/friends during the past 12 months (e.g., being “teased,” “hit,” “sexually assaulted,” “threatened,” and “excluded socially”). Five response options ranged from “not at all” to “more than 3 times a week” (α = 0.77). Perceived peer relationship stress was measured with one item, “In the past 12 months, how much stress have you experienced due to your relationship with peers?” (not at all true to very true).

In the school context, teacher abuse was measured with two items asking about the level of physical and verbal abuse incurred from teachers at school in the past 12 months (not at all to more than 3 times a week). The Spearman–Brown coefficient for teacher abuse was 0.71. Teacher support was measured with two items, “How much schoolteachers respect me in the past 12 months?” and “How much schoolteachers care for me in the past 12 months?” (not at all true to very true). The Spearman–Brown coefficient for teacher abuse was 0.84. School connectedness was measured by one item, “I have enjoyed going to school in the past 12 months” (not at all true to very true).

### 2.3. Data Analysis

In the first phase, descriptive analyses with frequency distributions and mean and standard deviation scores were estimated to examine the characteristics of social-ecological variables, CBV, and suicidal ideation. In the second phase, the Wald chi-square test was conducted to compare the estimates of CBV and suicidal ideation by sex and school level. Bivariate correlations between the variables were also examined. Then, a series of multivariate logistic regression analyses were performed to examine the association between CBV, socio-ecological variables, and suicidal ideation. The five models of the logistic regression analyses were included: (a) CBV and individual-level variables in Model-1, (b) family-level variables added in Model-2, (c) peer-level variables added in Model-3, (d) school-level variables included in Model-4, and (e) moderating effects of socio-ecological variables in Model-5. To determine if there were multicollinearity issues between family, peer, and school factors, variance inflation factors (VIF) and tolerance were tested in the proposed regression models. The VIFs for the variables did not exceed 3 and all tolerances of the variables were larger than 0.3, indicating that there were no issues with multicollinearity in the model [53]. All statistical analyses were conducted using STATA version 16 (StataCorp, College Station, TX, USA). 

## 3. Results

### 3.1. Descriptive Statistics and Correlational Analysis

As displayed in Table 1, 17.7% of students were victims of cyberbullying, and over 1 in 4 students (28.4%) thought about suicide in the past 12 months. Results show the sex differences in CBV (*χ*^2^ = 43.04, *p* < 0.001) and suicidal ideation (*χ*^2^ = 132.95, *p* < 0.001). More male adolescents (20.6%) were victims of cyberbullying than females (14.8%), while more female adolescents thought about suicide (34.4%) than did males (22.2%). Further, school-level difference was detected in CBV (*χ*^2^ = 30.80, *p* < 0.001). Middle school students (20.1%) reported a higher frequency of CBV compared with high school students (15.1%). Although more high school students thought about suicide (29.3%) than did middle school students (27.6%), no school-level difference was revealed in students’ suicidal ideation.

Table 2 depicts the correlations of the key study variables. All variables were correlated with each other. In particular, suicidal ideation was positively correlated with CBV, depressive symptoms, parental abuse and neglect, family dysfunction, peer victimization, perceived peer relationship stress, and teacher abuse, while it was negatively correlated with self-report physical health, self-esteem, parental support for autonomy, teacher support, and school connectedness.

### 3.2. Cyberbullying Victimization and Suicidal Ideation in Social-Ecological Contexts

Table 3 presents the results of multivariate logistic regression analyses investigating suicidal ideation associated with CBV and the social-ecological covariates among Korean adolescents. In Model-1, CBV and individual-level covariates were included. The overall model fits were significant (*χ*^2^ (6) = 1482.81, *p* < 0.001) and explained 17% of the variance. CBV was positively associated with suicidal ideation (OR = 1.65, *p* < 0.001). Older adolescents (OR = 0.89, *p* < 0.01) and male adolescents (OR = 0.76, *p* < 0.001) were less likely to report suicidal ideation than younger and female counterparts. Students with middle and high levels of GPA (OR = 0.83, *p* < 0.05; OR = 0.83, *p* < 0.01) and those with middle and high levels of SES (OR = 0.82, *p* < 0.05; OR = 0.73, *p* < 0.001) reported a lower risk of suicidal ideation compared with those in the low GPA and low SES groups. Good physical health (OR = 0.81, *p* < 0.001) and higher self-esteem (OR = 0.86, *p* < 0.001) were negatively associated with suicidal ideation, while greater depressive symptoms were positively associated with suicidal ideation (OR = 1.36, *p* < 0.001). 

Adding family factors in Model-2 increased the explained variance to 20% and showed significant overall model fits. Among the four family factors, parental abuse, parental support for autonomy, and family dysfunction were significantly associated with suicidal ideation. Parental abuse (OR = 1.22, *p* < 0.001) and family dysfunction (OR = 1.33, *p* < 0.001) were positively related to suicidal ideation, while parental support for autonomy (OR = 0.96, *p* < 0.01) was negatively related to suicidal ideation. For Model-3, peer victimization and perceived peer relationship stress were contained, and the overall model fits were significant and explained 22% of the variance. Both peer victimization (OR = 1.06, *p* < 0.01) and perceived peer relationship stress (OR = 1.19, *p* < 0.001) were positively associated with adolescent suicidal ideation. All the significant factors in Model-2 except SES remained significant for suicidal ideation in Model-3. Model-4 included school factors (teacher abuse, teacher support, and school connectedness), and the overall model fits were significant. Only school connectedness was associated with lower odds of suicidal ideation (OR = 0.85, *p* < 0.01). Students who perceived themselves to be well connected with school were less likely to have suicidal ideation.

### 3.3. Moderating Effect of Social-Ecological Contexts

**Figure 1:**
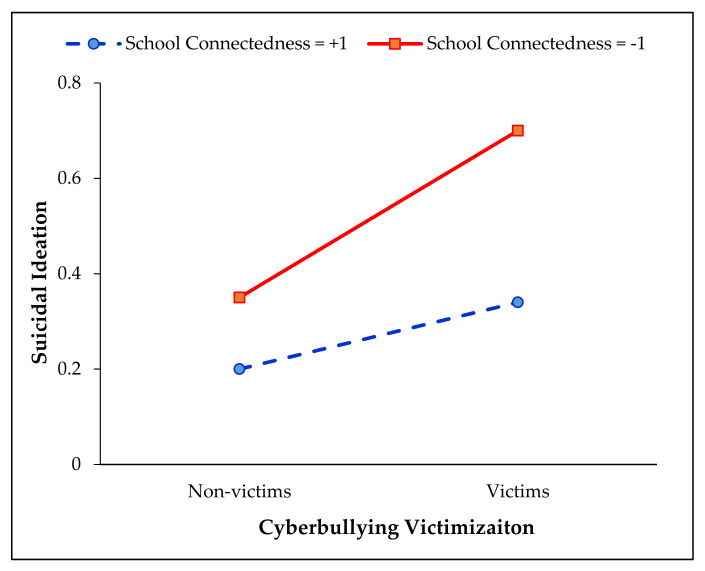
Moderating effect of school connectedness on the relationship between cyberbullying victimization and suicidal ideation. Simple slopes plot at low (–1 SD) and high (+1 SD) levels of school connectedness.

To test the moderating effects of social-ecological contexts, interactions between CBV and each family-, peer-, and school-level variable was added to the regression model (χ^2^ (29) = 1767.76, *p* < 0.001). As shown in the findings of Model-5 in Table 3, there was a significant interaction between CBV and school connectedness (OR = 1.27, *p* < 0.05). To further probe this interaction, follow-up tests of simple slopes were conducted for adolescents that were either high (+1 SD) or low (−1 SD) on overall school connectedness. Consistent with the healthy context paradox [46], the link between CBV and suicidal ideation was significantly weaker for adolescents with higher levels of school connectedness compared with those with lower levels of school connectedness (see Figure 1).

## 4. Discussion

The current study aimed to assess the relationship between CBV, social-ecological variables, and suicidal ideation among middle and high school students in South Korea. Consistent with previous research [12,13,41], findings from this study showed that being cyberbullied was positively associated with suicidal ideation in Korean adolescents.

In terms of the social-ecological model, at the individual level, age, male, GPA, SES, self-report physical health, and self-esteem were negatively associated with suicidal ideation, while depressive symptoms were positively associated with suicidal ideation. The current findings are in line with prior studies, indicating that adolescents’ demographic characteristics, social status, and physical and emotional condition are critical factors in understanding suicidal ideation [13,16,18,19]. In particular, younger and female adolescents were more likely to experience suicidal ideation than older and male counterparts [12,15]. This suggests that early prevention programs and gender-response interventions are required on the issue of suicidal ideation. Follow-up studies could consider investigating the differences between young and old adolescents and between male and female adolescents in their suicidal thoughts and attempts.

In the family context, consistent with the previous literature [22,24,29], the findings suggest that parental abuse and family dysfunction are significant risk factors, and parental support for child autonomy is a protective factor for adolescent suicidal ideation. However, parental neglect was not significantly related to suicidal ideation, which is inconsistent with previous studies [24,25]. This could be because although parental neglect may result in negative behavioral and psychological consequences, it does not directly relate to suicidal ideation. In a similar context, prior studies consistently indicated that parental support for autonomy protects adolescents against cyberbullying [54,55,56,57]. For example, adolescents with parents who use autonomy-supportive strategies were less likely to engage in cyberbullying behavior than adolescents with parents who use controlling strategies [49]. This study further explains that parental support for autonomy decreases reactance in adolescents and, in turn, also decreases participation in antisocial behaviors such as cyberbullying [54]. Therefore, it is required to enhance parental support on autonomy to deal with both cyberbullying behaviors and suicidal ideation among adolescents.

When peer factors were added in Model-3, both peer factors were significantly associated with adolescent suicidal ideation, which is consistent with previous research [7,31,33]. The findings imply that peer victimization and peer association are pivotal factors that exert important influences on Korean students’ behavioral health, especially in terms of suicidal ideation and attempts [58,59]. As adolescence is an essential transition between childhood and young adulthood, peer relationships/cohesion and peer victimization are the most important developmental predictors of suicidal behavior. As such, the development and implementation of programs that enhance peer association and pre-empt peer victimization would reduce suicidal behavior.

In the school context, in line with the empirical literature [32,35,43], school connectedness was found to be a protective factor for suicidal ideation among Korean students. The majority of Korean adolescents spend most of their time in school; it is where they learn, build relationships with their friends and teachers, and develop social-emotional skills. Therefore, students who do not feel a sense of belonging and connectedness to their school may be at increased risk of suicidal ideation and behavior. Unfortunately, few programs or interventions have highlighted the importance of the relationship between school connectedness and suicidal ideation and behavior for adolescents in South Korea. Additionally, strong school connectedness was found to buffer against the link between CBV and suicidal ideation. These findings complement existing investigations showing that school connectedness seems to be of substantial relevance to prosocial and positive psychological functioning. Indeed, a series of prior studies found school connectedness to confer resilience against suicidal ideation [46,47].

### 4.1. Implications

Previous studies have examined the relationship between CBV and suicidal ideation [12,13] and confirmed that the more CBV students experience, they are likely to report higher suicidal ideation [42]. However, these studies generally focused on the direct association between CBV and suicidal ideation and not on the moderating effects that could possibly affect this relationship to a varying degree. In this respect, the moderating effect of school connectedness between cyberbullying victimization and suicidal ideation was a meaningful finding in that it once again highlights the role of school in preventing cyberbullying as well as suicidal ideation based on the social-ecological model [60,61]. To mitigate the impact of cyberbullying and suicide on adolescents, various stakeholders from different settings should be aware of these problems. Regarding cyberbullying, adolescents themselves, who may be potential bullies, victims, or bystanders, should be aware of the negative consequences that cyberbullying brings upon victims, learn socially acceptable etiquette on the Internet, understand that revealing personal information online is very risky behavior, and be taught to use adequate coping strategies [62]. Moreover, since school connectedness had been found as a buffering variable that weakens the relationship between CBV and suicidal ideation, intervention at the school level should be made. For example, as school counselors and youth workers are easily accessible stakeholders for adolescents, they should be knowledgeable about the issue of cyberbullying so as to help cyber victims and cyberbullies with a strength-based approach and teach soft skills and social-emotional learning [63]. From a broader point of view, policymakers are also regarded as important stakeholders because they exert a strong influence on initiating and monitoring nationwide cyberbullying policies and educations [62]. Meanwhile, this study also determined that parental abuse, parental support for autonomy, and family dysfunction are significant factors associated with adolescents’ suicidal ideation. This implies that a positive relationship with parents reduces the risk of suicidal ideation and a negative relationship worsens the situation. Similar to this finding, previous studies have revealed a substantial discrepancy between adolescent-reported suicidal behavior and parents-reported suicidal behavior [64,65]. Most parents were unaware that their child had thought of or even attempted suicide. This indirectly implies that parental awareness is critical in reducing the risk of suicidal behavior among adolescents [65].

As mentioned above, there is an urgent need for suicide prevention and intervention strategies for Korean adolescents. Numerous efforts have already been made to prevent and treat suicide problems in South Korea by both government and non-governmental organizations [66]. However, the suicide rate of adolescents still has not been significantly reduced [67]. Moreover, some prior studies indicate that there are currently no well-established treatments shown to prevent or reduce suicidal ideation and behavior in youth [68,69]. Liu recommended a new suicide prevention program strategy for Korean adolescents that integrates national/societal involvement, school-based programs, and family-based programs [66]. That is, with a strong focus across individual, relationship, and community levels, suicide could be preventable. As this study’s findings also emphasized the diverse risk factors associated with CBV and suicidal problems, we suggest programs and counseling support to be regular and long-term rather than a one-off.

Currently, cyberbullying group counseling, prevention education, and media literacy education are being conducted at Internet addiction prevention counseling centers in South Korea [70]. However, these programs are mainly for preventive purposes and target all school-aged children rather than victims specifically. Therefore, programs specifically meant for victims of cyberbullying should be developed. Furthermore, mounting evidence supports that school-based cyberbullying programs and interventions are effective [71], and this study demonstrates that school connectedness is a buffer against suicidal ideation. However, school-based programs are generally lacking. As such, we propose that prevention and intervention programs in school settings must be considered to reduce cyberbullying and suicidal behavior and to promote students’ behavioral health.

### 4.2. Limitations

The current study has several limitations that should be noted. This is the first study to assess the association between CBV and suicidal ideation among a sample of Korean adolescents by considering social-ecological contexts. Given the promising findings of the study, further research utilizing a longitudinal design would be valuable. Despite existing evidence that warrants the predictive ability and relevance of single items measuring suicidal ideation [72], it would be good for future research to consider more holistic measures of suicidal ideation and behavior. Furthermore, this study used only suicidal ideation as the outcome of interest. Suicidal ideation, plans, and attempts should be considered distinctly. As such, it would be important to extend the current study by examining whether the association between cyberbullying and suicidal ideation would eventually lead to suicide attempts. Lastly, the measure of cyberbullying utilized in this study was to examine the context of cyberbullying and did not include items regarding types of digital use and devices (e.g., types of social media platforms). In South Korea, the increasing usage of social media and smartphones among adolescents may affect the risk of CBV and suicidality. Moreover, a collective type of bullying is commonly prevalent in South Korea [58]. Thus, it is necessary for future research to elaborate validating measures of cyberbullying for Korean youth.

## 5. Conclusions

Despite accumulating studies on the association of suicidal ideation with cyberbullying among adolescents, the social-ecological approach in the association between CBV and suicidal ideation among middle and high school students in South Korea has been undocumented. In addition, the moderating role of school connectedness in this association has not been fully understood. This study extended previous studies by exploring the relationship between CBV, multilevel social-ecological contexts, and suicidal ideation among a nationally representative sample of Korean adolescents. The findings highlight the importance of the association between being cyberbullied and suicidal ideation using a social-ecological approach, including individual-, family-, peer-, and school-level variables, and the potential for school connectedness to buffer cyber victims from developing suicidal ideation. Further research is warranted to prospectively examine the pathways or processes linking patterns of cyberbullying and suicidal behavior, which would help us to understand the vital changes taking place in developmental processes that are related to suicidal behavior among adolescent cyber victims. Prevention and intervention efforts to lessen the rates of CBV should deliberate the ubiquity of behavioral health concerns, including suicidal ideation, suicide attempts, and suicidality that may limit adolescents’ well-being and development.

## Figures and Tables

**Table 1:** Prevalence of cyberbullying victimization and suicidal ideation among Korean adolescents.

Variables	Total (N = 7333)	Male (N = 3608)	Female (N = 3725)		Middle School (N = 3775)	High School (N = 3558)	
	*n* (%)	*n* (%)	*n* (%)	*χ* ^2^	*n* (%)	*n* (%)	*χ* ^2^
1. Cyberbullying victimization	1294 (17.7)	743 (20.6)	551 (14.8)	43.04 ***	756 (20.1)	539 (15.1)	30.80 ***
Hearing profanity or insults	1066 (14.6)	645 (17.9)	421 (11.3)	64.18 ***	626 (16.6)	440 (12.4)	26.45 ***
Receiving threats	136 (1.9)	92 (2.6)	44 (1.2)	18.89 ***	89 (2.4)	47 (1.3)	10.86 **
Being made the subject of sexual jokes	186 (2.5)	97 (2.7)	89 (2.4)	0.68	112 (3.0)	74 (2.1)	5.87 **
Private matters being widely exposed	387 (5.3)	200 (5.6)	187 (5.0)	1.03	225 (6.0)	162 (4.6)	7.36 **
Experiencing exclusion during Internet use	125 (1.7)	51 (1.4)	74 (2.0)	3.57 *	93 (2.5)	32 (0.9)	26.79 ***
2. Suicidal ideation				132.95 ***			2.79
Yes	2074 (28.4)	798 (22.2)	1276 (34.4)		1034 (27.6)	1040 (29.3)	
No	5221 (71.6)	2790 (77.8)	2431 (65.6)		2716 (72.4)	2505 (70.7)	

* *p* < 0.05, ** *p* < 0.01, *** *p* < 0.001.

**Table 2:** Bivariate correlations of the key study variables.

Variables	1	2	3	4	5	6	7	8	9	10	11	12	13	14
1. Cyberbullying victimization														
2. Self-report physical health	−0.07 ***													
3. Depressive symptoms	0.04 ***	−0.28 ***												
4. Self-esteem	−0.08 ***	0.32 ***	−0.36 ***											
5. Parental abuse	0.21 ***	−0.08 ***	0.18 ***	−0.13 ***										
6. Parental neglect	0.13 ***	−0.12 ***	0.15 ***	−0.14 ***	0.28 ***									
7. Parental support for autonomy	−0.13 ***	0.17 ***	−0.19 ***	0.34 ***	−0.34 ***	−0.24 ***								
8. Family dysfunction	0.13 ***	−0.21 ***	0.31 ***	−0.27 ***	0.38 ***	0.25 ***	−0.37 ***							
9. Peer victimization	0.27 ***	−0.08 ***	0.13 ***	−0.09 ***	0.22 ***	0.22 ***	−0.11 ***	0.12 ***						
10. Perceived peer relationship stress	0.15 ***	−0.25 ***	0.45 ***	−0.27 ***	0.15 ***	0.12 ***	−0.17 ***	0.34 ***	0.18 ***					
11. Teacher abuse	0.15 ***	−0.04 ***	0.08 ***	−0.05 ***	0.23 ***	0.13 ***	−0.11 ***	0.10 ***	0.21 ***	0.03 *				
12. Teacher support	−0.09 ***	0.17 ***	−0.23 ***	0.24 ***	−0.12 ***	−0.10 ***	0.25 ***	−0.18 ***	−0.09 ***	−0.16 ***	−0.35 ***			
13. School connectedness	−0.09 ***	0.26 ***	−0.30 ***	0.39 ***	−0.08 ***	−0.12 ***	0.25 ***	−0.21 ***	−0.12 ***	−0.29 ***	−0.15 ***	0.38 ***		
14. Suicidal ideation	0.14 ***	−0.19 ***	0.39 ***	−0.28 ***	0.25 ***	0.14 ***	−0.21 ***	0.28 ***	0.13 ***	0.27 ***	0.08 ***	−0.16 ***	−0.22 ***	
M	0.18	1.99	3.30	7.85	0.90	0.31	8.82	0.63	0.47	0.83	0.72	4.00	1.90	0.28
*SD*	0.38	0.65	2.55	2.50	1.45	1.06	2.30	0.78	1.80	0.85	1.46	1.33	0.77	0.45
Min.–Max.	0–1	0–3	0–9	0–12	0–8	0–12	0–12	0–3	0–28	0–3	0–8	0–6	0–3	0–1

* *p* < 0.05, *** *p* < 0.001.

**Table 3:** Logistic regressions that predicted suicidal ideation by cyberbullying victimization and social-ecological variables.

Variable	Model-1	Model-2	Model-3	Model-4	Model-5
	OR (95% CI)	OR (95% CI)	OR (95% CI)	OR (95% CI)	OR (95% CI)
Cyberbullying victimization	1.65 (1.43–1.90) ***	1.42 (1.22–1.66) ***	1.30 (1.11–1.53) **	1.26 (1.07–1.48) **	1.35 (0.55–3.30)
Age	0.89 (0.83–0.95) **	0.92 (0.86–0.99) *	0.93 (0.86–0.99) *	0.92 (0.85–0.99) *	0.92 (0.85–0.99) *
Gender: male	0.76 (0.67–0.86) ***	0.72 (0.63–0.81) ***	0.73 (0.64–0.84) ***	0.73 (0.64–0.83) ***	0.73 (0.64–0.83) ***
School level: middle school	0.87 (0.69–1.08)	0.85 (0.67–1.08)	0.84 (0.66–1.07)	0.86 (0.68–1.10)	0.86 (0.68–1.10)
School GPA					
High	0.83 (0.72–0.95) **	0.84 (0.73–0.97) *	0.84 (0.73–0.97) *	0.85 (0.73–0.98) *	0.84 (0.73–0.98) *
Middle	0.83 (0.71–0.97) *	0.82 (0.69–0.97) *	0.81 (0.69–0.96) *	0.82 (0.69–0.97) *	0.82 (0.69–0.97) *
SES					
High	0.73 (0.61–0.86) ***	0.83 (0.69–0.99) *	0.84 (0.70–1.00)	0.83 (0.69–0.99) *	0.83 (0.69–0.99) *
Middle	0.82 (0.69–0.97) *	0.94 (0.79–1.13)	0.96 (0.80–1.15)	0.95 (0.79–1.14)	0.95 (0.79–1.14)
Self-report physical health	0.81 (0.74–0.89) ***	0.84 (0.76–0.93) **	0.87 (0.78–0.96) **	0.87 (0.79–0.97) *	0.88 (0.79–0.97) *
Depressive symptoms	1.36 (1.33–1.40) ***	1.34 (1.30–1.37) ***	1.31 (1.27–1.35) ***	1.31 (1.27–1.35) ***	1.31 (1.27–1.35) ***
Self-esteem	0.86 (0.84–0.88) ***	0.88 (0.85–0.90) ***	0.88 (0.86–0.98) ***	0.89 (0.87–0.92) ***	0.89 (0.87–0.92) ***
Parental abuse		1.22 (1.16–1.27) ***	1.21 (1.16–1.27) ***	1.21 (1.15–1.26) ***	1.20 (1.13–1.26) ***
Parental neglect		1.03 (0.97–1.09)	1.02 (0.96–1.08)	1.03 (0.97–1.10)	1.03 (0.96–1.11)
Parental support for autonomy		0.96 (0.93–0.99) **	0.96 (0.93–0.98) **	0.96 (0.93–0.99) *	0.96 (0.93–0.99) *
Family dysfunction		1.33 (1.22–1.45) ***	1.29 (1.18–1.41) ***	1.29 (1.18–1.40) ***	1.30 (1.18–1.43) ***
Peer victimization			1.06 (1.02–1.10) **	1.07 (1.03–1.11) **	1.06 (0.99–1.12)
Perceived peer relationship stress			1.19 (1.10–1.29) ***	1.17 (1.08–1.27) ***	1.17 (1.07–1.28) **
Teacher abuse				1.02 (0.97–1.07)	1.03 (0.98–1.09)
Teacher support				0.98 (0.93–1.03)	1.01 (0.95–1.07)
School connectedness				0.85 (0.78–0.94) **	0.81 (0.73–0.90) ***
Cyberbullying victimization × school connectedness					1.27 (1.02–1.58) *
χ^2^ (*df*)	1482.81 (11) ***	1710.70 (15) ***	1741.72 (17) ***	1759.65 (20) ***	1767.76 (29) ***
Pseudo *R*^2^	0.17	0.20	0.21	0.22	0.22

* *p* < 0.05, ** *p* < 0.01, *** *p* < 0.001. OR = odds ratio; CI = confidence interval. Reference groups for gender, school level, SES, and school GPA are female, high school, low SES, and low GPA, respectively.

## Data Availability

The KCYRS data are available in the NYPI Youth and Children Data Archive, https://www.nypi.re.kr/archive/mps (accessed on 15 December 2020).
